# Bulk tumour cell migration in lung carcinomas might be more common than epithelial-mesenchymal transition and be differently regulated

**DOI:** 10.1186/s12885-018-4640-y

**Published:** 2018-07-06

**Authors:** Martin Zacharias, Luka Brcic, Sylvia Eidenhammer, Helmut Popper

**Affiliations:** Diagnostic and Research Center, Institute of Pathology, Medical University of Graz, Neue Stiftingtalstraße 6, Graz, 8036 Austria

**Keywords:** Lung cancer, Bulk migration, Squamous cell carcinoma, Adenocarcinoma, Protein expression, Twist, Mad, Tks5, Cadherin

## Abstract

**Background:**

Epithelial-to-mesenchymal transition (EMT) is one mechanism of carcinoma migration, while complex tumour migration or bulk migration is another - best demontrated by tumour cells invading blood vessels.

**Methods:**

Thirty cases of non-small cell lung carcinomas were used for identifying genes responsible for bulk cell migration, 232 squamous cell and adenocarcinomas to identify bulk migration rates. Genes expressed differently in the primary tumour and in the invasion front were regarded as relevant in migration and further validated in 528 NSCLC cases represented on tissue microarrays (TMAs) and metastasis TMAs.

**Results:**

Markers relevant for bulk cancer cell migration were regulated differently when compared with EMT: Twist expressed in primary tumour, invasion front, and metastasis was not associated with TGFβ1 and canonical Wnt, as Slug, Snail, and Smads were negative and β-Catenin expressed membraneously. In the majority of tumours, E-Cadherin was downregulated at the invasive front, but not absent, but, coexpressed with N-Cadherin. Vimentin was coexpressed with cytokeratins at the invasion site in few cases, whereas fascin expression was seen in a majority. Expression of ERK1/2 was downregulated, PLCγ was only expressed at the invasive front and in metastasis. Brk and Mad, genes identified in Drosophila border cell migration, might be important for bulk migration and metastasis, together with invadipodia proteins Tks5 and Rab40B, which were only upregulated at the invasive front and in metastasis. CXCR1 was expressed equally in all carcinomas, as opposed to CXCR2 and 4, which were only expressed in few tumours.

**Conclusion:**

Bulk cancer cell migration seems predominant in AC and SCC. Twist, vimentin, fascin, Mad, Brk, Tsk5, Rab40B, ERK1/2 and PLCγ are associated with bulk cancer cell migration. This type of migration requires an orchestrated activation of proteins to keep the cells bound to each other and to coordinate movement. This hypothesis needs to be proven experimentally.

**Electronic supplementary material:**

The online version of this article (10.1186/s12885-018-4640-y) contains supplementary material, which is available to authorized users.

## Background

Migration and invasion into the stroma is a requisite for cancer metastasis. Two mechanisms have been described in the last decade, namely epithelial to mesenchymal transition (EMT) and complex tumour cell migration. In EMT, as primarily described in experimental studies, the tumour cells downregulate adherence proteins, lose contact to cells, and change to a mesenchymal phenotype, often by exchanging keratin for vimentin or α-actin [1, 2]. These cells develop invadipodia and move as individual cells or in small groups. Several genes have been identified as EMT drivers, namely snail family transcriptional repressor 2 (Slug), twist family bHLH transcription factor 1 (TWIST), Zinc finger E-box binding homeobox 1 (ZEB1), nuclear-translocated β-Catenin, transforming growth factor beta (TGF-β), and the frizzled class receptor family (frizzled) [3–10]. Among examples for EMT in pulmonary carcinomas are small cell carcinomas (SCLC), micropapillary adenocarcinomas, and pleomorphic carcinomas (PC), whereas in the majority of adenocarcinomas (AC), squamous cell carcinomas (SCC), and large cell carcinomas (LC), EMT seems to be rare. In complex tumour migration, the cells form cell clusters and disconnect from the main tumour [11]. EMT has been thought to be essential for metastasis. However, recent findings have challenged this view and proposed complex tumour cell migration as an alternative. Some have called this hybrid EMT, and it has even been experimentally shown that cells can metastasize without changing to a mesenchymal phenotype, called the epithelial migration type [12–15]. Although SCLC as well as micropapillary adenocarcinomas move in small cell clusters of 3–5 cells or as single cells and invade the primary as well as the metastatic organ site diffusely, in the majority of cases, they do not lose their epithelial phenotype. Neither loses cytokeratin – thus, they are a part of hybrid-EMT. Pulmonary sarcomatoid carcinomas, especially spindle cell carcinomas, often occur in the primary tumour with a mesenchymal phenotype, expressing α-actin instead of keratin. They may continue doing this during metastasis, or they may revert back to an epithelial phenotype at the metastatic site.

**Fig. 1:**
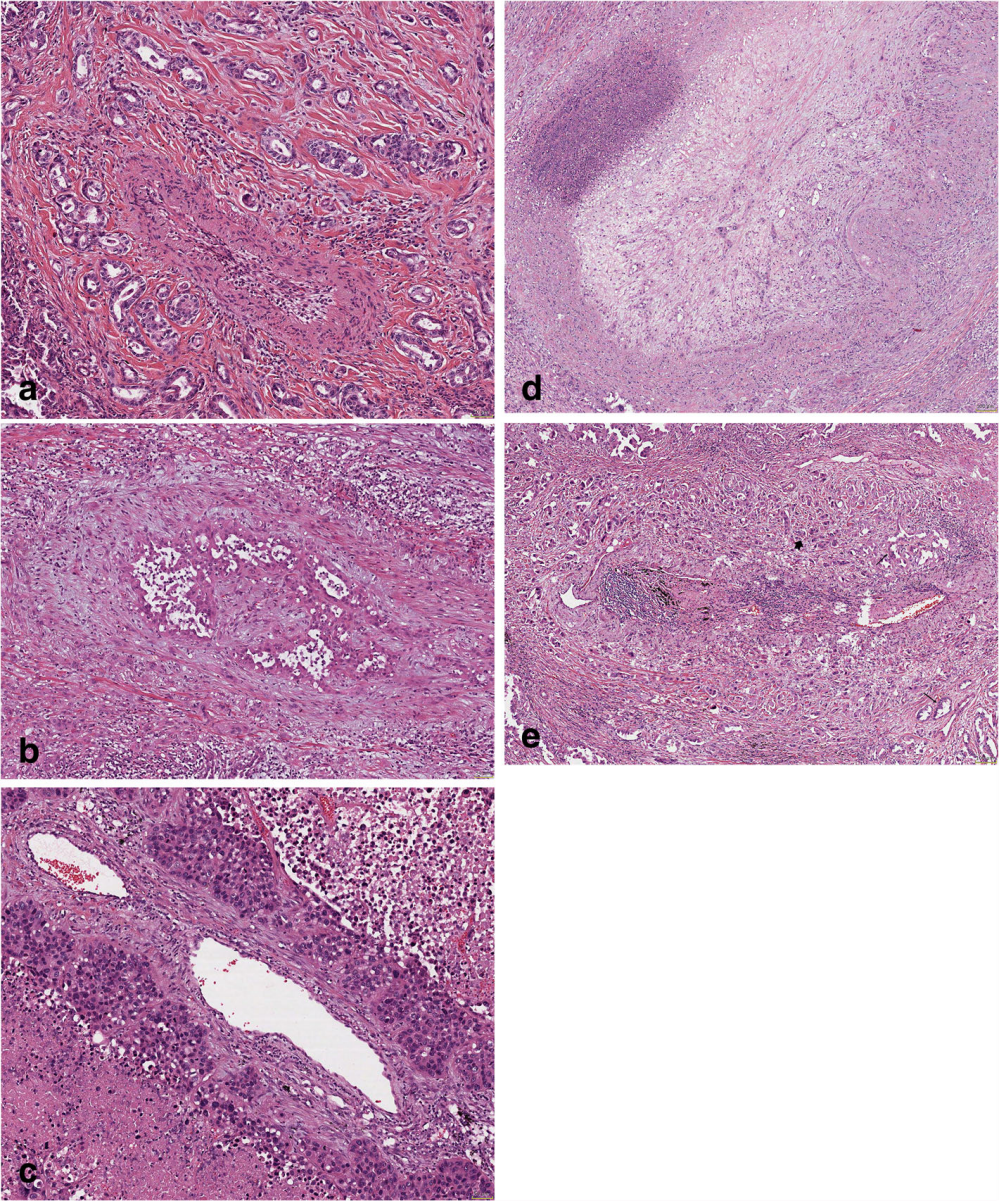
Photomicrographs illustrating different cases with bulk cancer cell migration (**a**, **b**, **c**), mixed bulk and EMT migration (**e**), and classical EMT (**d**); in all of them carcinomas cells invade the vascular wall, in some into the lumen; **a**-**c** adenocarcinomas, D squamous cell carcinoma. H&E, magnification bars 50 μm (**a**, **b**) and 25× (**c**, **d**)

Due to the fact that vascular invasion is a poor prognostic factor [16], we usually evaluate carcinomas for vascular invasion. We identified well-formed AC and SCC cell clusters within the vascular walls, still forming differentiated structures such as glands or plate-like sheets. Because these tumour cells are disconnected from the main tumour, they must have migrated in these complexes or bulks (Fig. 1a, b, c). These bulks do not lose their keratin cytoskeleton and generally do not express vimentin or other markers of EMT, indicating that bulk cell migration is an alternative mechanism, for which the driving genes have not been identified. In Drosophila wing border cell migration, four key genes have been identified as being related to this complex cell movement: receptor of activated C kinase (Rack1), brinker (Brk), mother against dpp (Mad), and saxophone (SAX) [17].

As a first step, we aimed to explore the frequency of bulk migration in AC and SCC, to evaluate molecules which might be associated with migration, invasion, and metastasis using immunohistochemistry. We are aware of limitations of this study: morphology provides a snapshot at a certain stage of development of a carcinoma, but cannot provide insight into dynamics of migration. However, we can compare expression patterns in the primary tumour with migrating and metastatic cells, which is not possible in a cell culture system. A morphological analysis can provide necessary information on which to base an experimental design.

## Methods

Thirty cases of AC and SCC were retrieved from the Lung Archive, all characterized by vascular invasion into pulmonary arteries or veins. The selected cases required having the central tumour, the invasion front to lung or pleura, and the vascular invasion on the same tissue slide to compare staining patterns side by side (Fig. 1; SETstudy). With a literature search, several molecules were identified as being associated with migration and invasion. Antibodies for these molecules were purchased and tested for their specificity. Dilution and pre-treatment was adapted when necessary (for details, see Table 1). As the study set had a rather small number of cases, we aimed to validate the importance of the markers in a larger cohort of NSCLC: tissue microarrays (TMA) were chosen which contained a total of 325 AC, 142 SCC, and 61 LC (validation set for immunohistochemistry; TMAval); out of these cases, whole tissue sections from 115 cases of SCC and 117 cases of AC were randomly chosen for the evaluation of bulk and/or EMT migration (SETval). To evaluate the importance of the markers for metastasis, another TMA, composed of 45 cases of primary tumour and up to three corresponding metastasis (TMAmets), was chosen.

**Table 1:** Antibodies used in this study including clone, dilution, and pretreatment

Marker	Company, clone	Dilution	Pretreatment	Visualization
Brk	Biosource	1:200	CC1mild	UV/DAB
CdC42	Santa Cruz	1:50	MW6,0	ENV/DAB
Connexin 43/GJA1	Novusbio	1:500	CC1mild	UV/DAB
CXCR1	Abcam	1:200	MW9,0	ENV/DAB
CXCR2	Abcam	1:200	MW9,0	ENV/DAB
CXCR4	Abcam 124824	1:500	retrievalDAKO WB	HRB-DAB
Ecadherin	Dako	Ready to use	high ph	Omnis Flex
Ncadherin (5D5)	Abcam	1:500	CC1mild	UV/DAB
ERK1/2 p44/42MAPK	Cell signaling	1:100	MW Tris	ENV/DAB
Focal Adhesion kinase FAK	Abcam	1:1000	MW6,0	ENV/DAB
Fascin 1	Chem	1:500	MW6,0	CM/AEC
Integrin-linked ILK phosphoS246	Abcam	1:100	CC1mild	UV/DAB
Mad	Novusbio	1:100	CC1mild	UV/DAB
PI3K p110α	Cell signaling	1:50	MW9,0	CM/AEC
PLCγ	Abcam	1:100	MW9,0	ENV/DAB
Rab40B	Proteintech	1:200	MW6,0	ENV/DAB
Rack1	Spring	1:100	CC1mild	UV/DAB
RhoA	Abcam	1:200	MW6,0	ENV/DAB
SARI/BATF2 (MGC20410)	Novusbio	1:200	CC1mild	UV/DAB
SAX	Abcam	1:200	CC1mild	UV/DAB
Slug	Novusbio	1:100	CC1mild	UV/DAB
SMARCA4/BRG1	Abcam	1:200	CC1mild	UV/DAB
Snail	Santa Cruz	1:50	MW6,0	ENV/AEC
pSRC phosphoS75	Abcam	1:100	CC1mild	UV/DAB
TGF1β	Santa Cruz	1:50	CC1mild	UV/DAB
Tks5	Novusbio	1:75	MW6,0	ENV/DAB
Twist(10E4E6)	Novusbio	1:50	MW6,0	ENV/DAB
Vim	Dako	Ready to use	low ph	Omnis Flex
YAP phospho127	Abcam	1:300	CC1mild	UV/DAB
ZEB1/ARBE6	Abcam	1:200	CC1mild	UV/DAB
ZFPL1	Abcam	1:200	CC1mild	UV/DAB

Each case in the TMA was represented by at least 3 cores of tumour tissue and 1 core of normal lung tissue or metastatic site. Immunohistochemistry in TMAval/TMAmets was performed for those markers, which showed a different staining pattern in the study set between the central tumour and the two invasion sites (front and/or vascular invasion). Three pathologists (MZ, LB, HP) evaluated the slides independently. All cases were discussed and re-evaluated together in cases of discordant scores.

## Results

### Study set

Twenty-eight out of 30 carcinomas showed bulk cancer migration, with tumour bulks also seen in the blood vessel walls (Fig. 1a-c). One case showed a typical EMT pattern (Fig. 1d), and in another case (Fig. 1e), a hybrid pattern was seen with both EMT and bulk migration. A cancer cell bulk was defined by a minimum of 15 cells, and in general, the number of cells forming a bulk was > 30. An EMT migration pattern was defined as single cell or small cell clusters of no more than 5 cells. A mixed pattern was defined when both types were present. ACs were either acinar, papillary, or solid subtypes, no micropapillary or cribriform types. SCC were well to moderately differentiated (Additional file 1: Table S1).

**Fig. 2:**
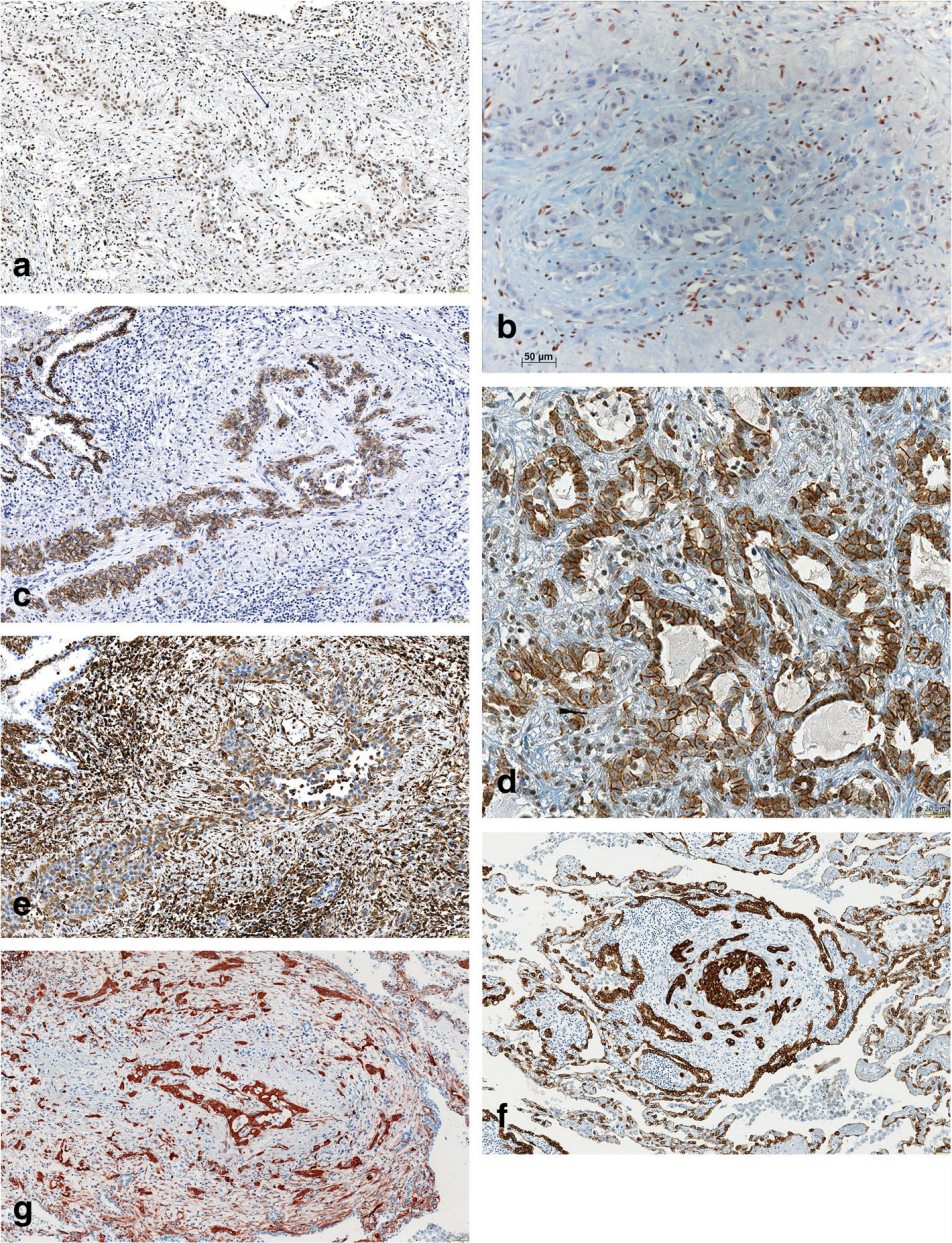
Immunohistochemistry in an area of vascular invasion; **a**) Twist, one arrow pointing to the carcinoma complexes (left), the other to the vascular wall (top), **b**) ZEB1, tumor cell nuclei are all negative; note the positively stained stroma cells, **c**) E-Cadherin, the tumour bulks are stained within the vascular wall, **d**) N-Cadherin, positively stained acini in the invasion front, **e**) vimentin, compare light staining of tumour cells and strong staining of the stroma cells, vimentin positive (single arrow) and negative (double arrow) tumour cells, **f**) cytokeratin, **g**) Fascin; magnification bars 50 μm (**a**-**c**, **e**-**g**), and 20 μm (**d**), respectively

All carcinomas demonstrated nuclear positivity for Twist (30/30), there was no difference between invasion and vascular invasion, and the main tumour. Staining intensities ranged from moderate to strong (Fig. 2a), and in some cases, in addition to nuclear, cytoplasmic staining was seen as well. Zinc finger E-box binding homeobox 1 (ZEB1), a gene under the control of Twist and known as the most important mediator for EMT, was negative in all cases (Fig. 2b), except for one pure EMT case.

All carcinomas stained for E-Cadherin (E-Cad) in a membranous pattern (Fig. 2c). The intensity varied: in 24 tumours, staining was less intense at the invasion and vascular invasion front compared to the central tumour; in 8 cases, a loss of staining was seen in some cells within the tumour bulk. N-Cadherin (N-Cad) was positive in all carcinomas, and staining intensity varied, with 9 cases strongly stained, and all others being less intense (Fig. 2d). All of these cases coexpressed E-Cad. In pre-tests, βCatenin showed membraneous staining. As there was also no loss of E-Cad, we did not evaluate the canonical Wnt family member(Wnt)-Frizzeld-βCatenin pathway. Vimentin was negative in 21/30 carcinomas. In 9 carcinomas within the vascular walls and at the invasion front, several tumour cells were positively stained for vimentin but still connected to the main tumour cell bulk. Vimentin-positive cells were absent in the central tumour, and, within the bulk, positive cells were concentrated at the periphery (Fig. 2e). All tumour cells, including vimentin-positive ones, were positive for cytokeratin (Fig. 2f). When comparing vimentin and E-Cad staining, it turned out that vimentin-positive tumour cells showed loss or reduction of E-Cad positivity.

Fascin antibodies stained 19 cases of central tumour and invasive front (Fig. 2g), in 7 additional cases, only the invasion site was positive. In 2 carcinomas the central tumour showed minimal focal staining, and only 2 cases were completely negative. Staining intensity was more pronounced at the invasive front and the vascular invasion compared to the central part of the carcinoma. All cases positive for vimentin also showed positive staining for fascin, but with less intensity.

**Fig. 3:**
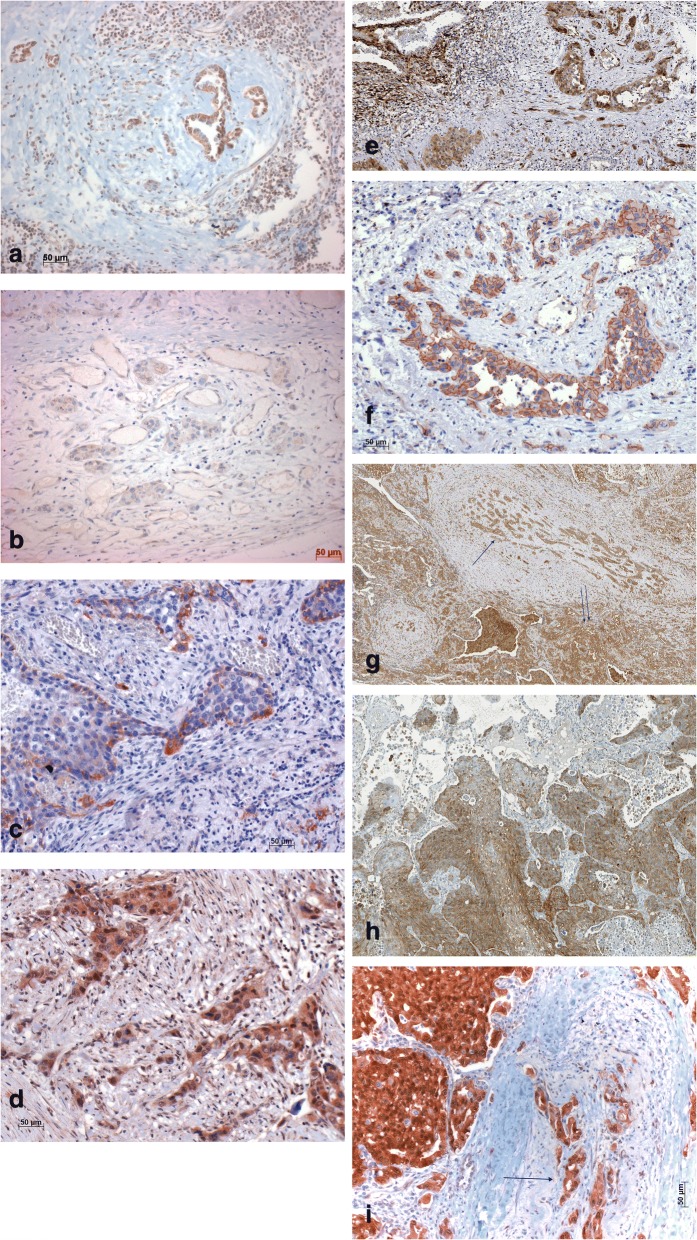
Immunohistochemistry for different markers associated with migration. **a**) Mad, **b**) Brk, **c**) Tks5, **d**) Rab40B, **e**) pERK1/2, **f**) PLCγ, **g**) RhoA, tumour centre (double arrow), vascular invasion (single arrow), **h**) connexin43, **i**) YAP1, arrow points to vascular invasion; magnification bars 50 (**a**-**f**, **h**-**i**) and 100 μm (**g**), respectively

Mother against dpp (Mad) and Brinker (Brk) were more intensely positive at the invasion site in 23/30 carcinomas (Fig. 3a). In 4 additional cases, only single positive tumour cells were found. Brk was focally positive in 15 tumours (Fig. 3b). All cases positively stained for Brk were also positive for Mad. Staining was nuclear as well as cytoplasmic. Saxophone (SAX) and receptor of activated C kinase (Rack1), the two other proteins associated with Drosophila wing border cell migration, were negative.

Staining for the two invadipodia-inducing proteins Rab40B (member of RAS oncogene family) and Tks5 (scaffold protein TKS5) showed positivity exclusively at invasion sites (Fig. 3c) in 21/30 cases (Tks5). In 11 cases Rab40B was coexpressed with Tks5 (Fig. 3d). All Tks5-positive cases were also positive for Mad.

Extracellular regulated MAP kinase (pERK1/2) was positive in the central areas of 10 carcinomas, and in 6 of them the invasion front was positive as well, however with lower intensity compared to the main tumour (Fig. 3e). In 16 carcinomas positivity was confined to the invasion front and vascular invasion, leaving the central tumour unstained; in one case, only the pleural invasion was positive. Phospholipase C gamma (PLCγ) staining was associated with stromal and pleural invasion in 22/30 cases - the primary tumours were negative in all cases (Fig. 3f). Tumour cell complexes within blood vessels (wall or lumen) also expressed PLCγ. In contrast, Ras homolog family member A (RhoA) was less intensely stained in the invasion front compared to the central tumour area in 27/30 cases (Fig. 3g). Cell division cycle 42 (CdC42) was focally positive in 5/30 cases, especially at the invasion front, and it was distributed randomly in the central tumour and invasion front in 2 cases.

Connexin43, an adhesion molecule for homing to lung endothelia, was positive in 20/30 carcinoma (Fig. 3h). These were most often scattered cells within the central as well as in the invasive carcinoma, being more numerous at the invasion site. C-X-C motif chemokine receptor 4 (CXCR4) was positive in 9 cases, in 7 of them very focally in a small number of tumour cells. There was no difference between the tumour centre and the invasion. CXCR2 was only focally positive in 11/30 carcinomas. CXCR1 was positive in all carcinomas, and no difference was found between central and invasive cells. The reaction pattern was focal with negative cells present in all cases. Focal adhesion kinase (FAK) was focally positive in 15 tumours, a more intense staining was seen in tumour centres compared to invasion fronts. A nuclear staining was seen for Integrin-linked kinase (ILK) in 10 cases, and in 8 additional cases, positivity was confined only to mitotic cells. Areas of invasion were constantly negative. Phosphorylated SRC proto-oncogene tyrosine kinase (pSRC) was positive in 6 cases; in 4 of them, positivity was seen exclusively in mitotic cells, and only 2 had a more random nuclear positivity. There was no correlation to invasion.

Antibodies for Slug, TGFβ1, and basic leucine zipper ATF-like transcription factor 2 (SARI) reacted negatively in all carcinomas. 13/30 cases were minimally positive for Snail, but only in a small number of cells. SWI/SNF related, matrix-associated, actin-dependent regulator of chromatin subfamily A member 4 (SMARCA4) was positive in all cases. There was no difference when the tumour centre, the invasion front and vascular invasion were compared. Yes-associated protein 1 (YAP1) was positive in 29/30 cases, the invasion site always being less intensely stained (Fig. 3i). In 21 cases, staining was nuclear as well as cytoplasmic. Zinc finger protein like 1 **(**ZFPL1**)** was positive in 8/30 tumours; however, in 7 of them, only few predominantly keratinized SCC cells stained; only one carcinoma was diffusely positive.

#### Validation set (SETval, TMAval)

**Fig. 4:**
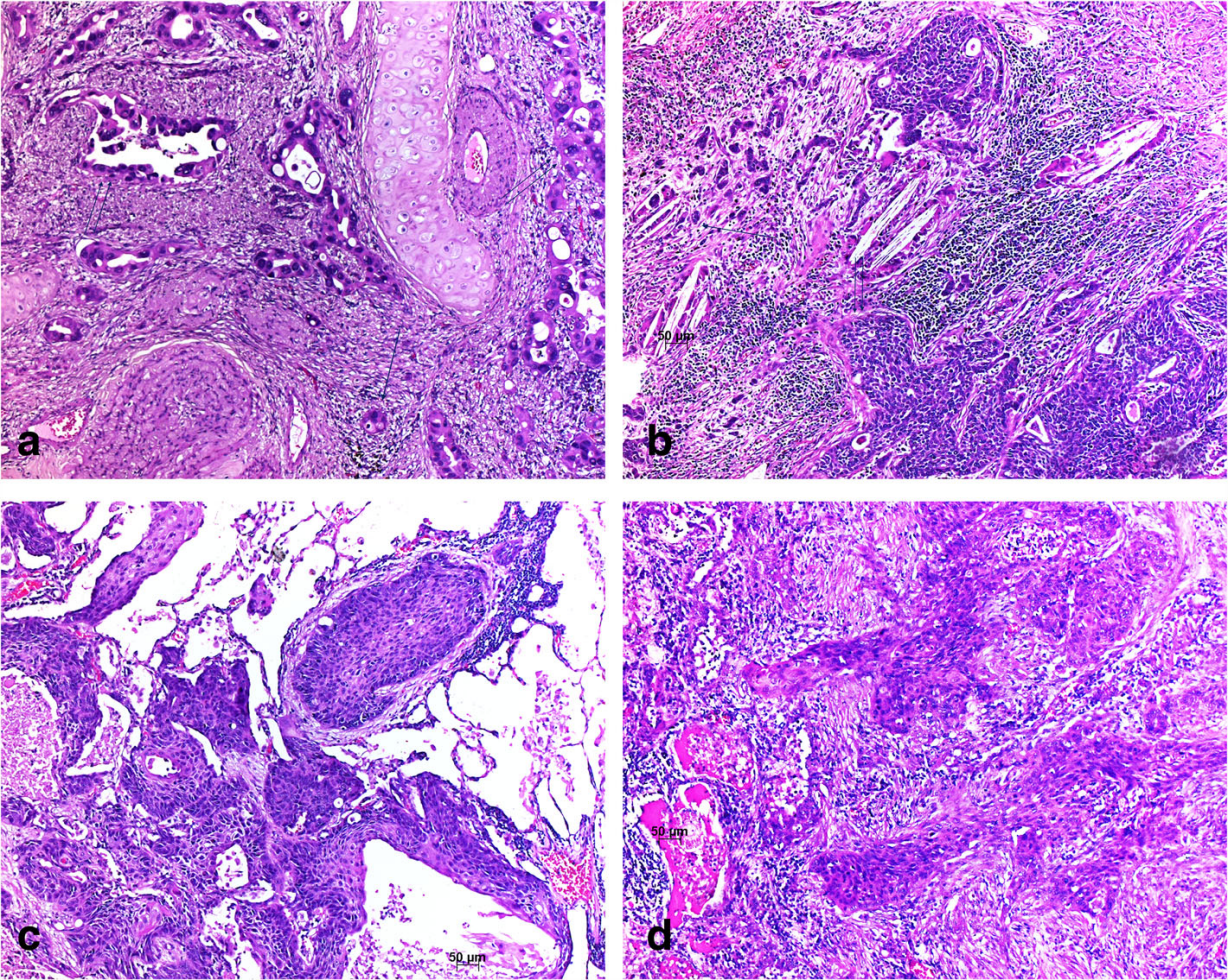
Examples from the evaluation set (SETval) are shown. **a** and **b** are examples of mixed bulk and EMT migration. Double arrows show bulk and single arrows EMT type migration; **c** and **d** are examples of bulk migration only; **a** and **c** acinar and solid adenocarcinomas, **b** and **d** squamous cell carcinomas; magnification in all cases × 100

From the validation set, 82 cases of SCC and 83 AC showed bulk migration, 2 SCC and 1 AC cases showed EMT migration pattern, and 31 SCC and 33 AC cases showed a mixed bulk and EMT migration pattern (SCC: 71, 27, 2% respectively, AC: 70, 28, < 1% respectively; Fig. 4a-d). Mixed bulk and EMT migration is defined by tumour bulks and single cell infiltration either coexisting in one area, or separately.

When looking up the staining patterns of SCC, large cell (LC), and AC on the TMAval, the following was observed (Table 2): E-Cad staining was lost in 1.2% of AC, 0.8% of SCC, and 8.3% of LC. Strong membranous staining was observed in 17.8% of AC, 43.5% of SCC, and 30% of LC. N-Cad was positive in all AC and SCC, and only negative in 3.3% of LC. Strong expression was seen in AC, SCC, and LC in 21.8, 12.1, and 13.3%, respectively. High percentages of positive cases were seen for Mad, and low percentages for Vimentin, whereas Twist was positive in half of the AC cases, and much higher in SCC and LC. Strong expression for pERK was seen in AC, SCC, and LC in 21.4, 13.2, and 33.3% respectively, while negative reactions were seen in 31.9% (AC), 45.6% (SCC), and 8.3% of LC cases respectively. Tks5 and PLCγ was expressed in more than half of all cases of AC, and SCC, whereas negative in LC in 63.9%.

**Table 2:** Expression of markers associated with migration in tissue microarrays for adenocarcinomas (325 cases), squamous cell carcinomas (142 cases), and large cell carcinomas (61 cases). For ECadherin, NCadherin, and pERK the percentages of negative as well as strong expression is given, for Mad, Twist, Vim, PLCγ, and Tks5 only positive reactions are recorded. For Connexin 43 a mean and standard deviation as well as the range is given

	Connexin	ECad	NCad	Mad	Twist	Vim	pERK	PCLγ	Tks5
AC	Mean 18.32 ± 24.83 Range 0–96	1.2% negative 17.8% strong positive	All cases positive, 21.8% strong positive	82.2% positive	50.7% positive	6.4% positive	31.9% negative 21.4% strong positive	53.1% positive	42.1% positive
SCC	Mean 53,12 ± 29.52 Range 0–90	0.8% negative 43.5% strong positive	All cases positive, 12.1% strong positive	81.0% positive	83.8% positive	15.2% positive	45.6% negative 13.2 strong positive	53.7% positive	73.2% positive
LC	Mean 30.38 ± 29.14 Range 0–90	8.3% negative 30.0% strong positive	3.3% negative 13.3% strong positive	80.0% positive	78.4% positive	47.6% positive	8.3% negative 33.3% strong positive	65.0% positive	36.1% positive

### Metastasis set (TMAmets)

The TMA with combined primary tumour and metastasis tissue was tested for Connexin, Tks5, MAD, TWIST, vimentin, PLCγ, and CXCR4. In the TMAmets brain and extrathoracic lymph nodes metastasis were predominantly present, while adrenal glands, liver, skin, and bone appeared less frequently. There was a good concordance of positively stained carcinoma cells in the primary tumour and the metastasis for MAD, connexin43, CXCR4, and PLCγ, whereas TWIST, vimentin, and Tks5, were significantly more highly expressed in metastasis (Table 3). However, a comment is needed on vimentin: in general, expression was restricted to few cases (19 metastasis, only 3 corresponding primary tumours), which makes statistical significance questionable (Additional file 2: Table S2).

**Table 3:** Frequency of positivity of carcinoma cells for connexin43, MAD, Twist, vimentin, PCLγ, Tks5, and CXCR4 in paired primary tumors and metastasis of predominantly AC and SCC

Protein	Primary tumor (%)	Metastasis (%)	Significance
Connexin 43	28.8 ± 34.2	25.4 ± 29.9	*p* 0.4
MAD	38.8 ± 30.1	32.2 ± 21.9	*p* 0.3
TWIST	23.8 ± 25.7	52.6 ± 27.6	*p* < 0.0001
Vimentin	2.1 ± 6.6	18.3 ± 30.7	*p* 0.005
PLCγ	17.6 ± 18.9	29.8 ± 20.1	*p* 0.02
Tks5	5.1 ± 6.3	14.3 ± 18.8	*p* 0.008
CXCR4	6.2 ± 12.1	4.7 ± 12.3	*p* 0.4

## Discussion

Migration of carcinoma cells is a complex process which requires modification of the adhesion to their neighbouring cells, formation of invadipodia, disconnection from the primary tumour, development of sensory capacity for oxygen tension and pH gradient, reading adhesion molecules present on matrix proteins to gain orientation (fibronectin, collagens, etc.), and creating energy for movement – all depending on the initiation of a multi-layered genetic program.

Here, we describe bulk cancer cell migration, also called complex movement or hybrid EMT [13, 18], as the predominant mode of migration in pulmonary squamous and adenocarcinomas. Carcinomas even retained their acinar, plate-like, or sheet formation. Our findings are based on a morphological analysis which provides a comparison of the primary tumour, migrating cells, and metastasis. If carcinoma complexes are seen within the vascular wall and lumen, it is very likely that the cells migrated there in the same bulk. Vascular invasion is therefore the optimal site for investigating the mode of migration. A comparison of expression of molecules between the primary tumour and the bulk cells might provide a first insight into which of these factors might be responsible for migration and allow a hypothesis to be formed. Dynamics of this process, such as up- and downregulation of molecules involved in bulk migration, cannot be evaluated on histology, but our findings can be used to create an experimental model.

### Proteins associated with bulk cancer migration

In bulk migration, there is no mesenchymal transition, as the tumour cells retained cytokeratin and E-Cad, although the latter downregulated at the invasion front. N-Cad was induced in all carcinomas more intensely stained at the invasion front, but always coexpressed with E-Cad. Although Twist was active in all tumours, its regulation was not controlled by TGFβ1 [19]. In contrast to EMT, Twist did not induce loss of E-Cad [1], and another EMT mediator, ZEB1 [20], was negative. Twist has been described as inducing Snail via the neurotrophic receptor tyrosine kinase B, yet in our cases, Snail was expressed in few cases and in single cells only [21]. However, Twist might have induced TAZ/Yap in all cases with bulk migration [22]. As TGFβ1 was negative in all carcinomas, downstream genes such as Slug and Smads’ were negative, too, and β-Catenin was regularly located at the cell membranes [20], ruling out any role for the canonical SARI-GSK3-βCatenin and Wnt-Frizzeld pathways to play in bulk migration. Interestingly, Twist was expressed highly in metastasis, pointing to an important function for metastasis as well. Twist most likely is not responsible for the upregulation of vimentin, as it was also expressed in vimentin-negative carcinomas. The loss of SARI (suppressor of AP-1 or BATF2) in all tumour cases also did not suppress E-Cad [23]. However, loss of SARI might have contributed to the expression of vimentin, being a vimentin suppressor [23].

In EMT, the exchange of cytokeratin for vimentin is explained by the fact that vimentin gives carcinoma cells more flexibility to change cell shape and thus adapt for migration better. E-Cad contributes to tight binding of carcinoma cells. Our finding of tumour cells coexpressing vimentin and cytokeratin at the bulk border and of reduced expression of E-Cad in these bulks (staining intensity equivalent to decreased receptor density) probably results in more freedom of movement while still keeping the cell together [24]. It might be speculated that the vimentin-expressing cells could act as ‘bulk officers’, coordinating movement, which is supported by recently published findings [25]. As this only happened in a minority of tumours, another mechanism such as fascin might replace vimentin in this function, which fits nicely with our results [26].

Fascin an actin-binding protein, was upregulated in tumour centres, but more intense at the invasion front. It might be another factor providing cross-talk within bulk cells. Fascin is crucial for filopodia formation [27]. Interestingly, expression of fascin was associated with expression of TAZ/YAP, which confirms a previous report linking YAP to migration [28]. Two of the proteins responsible for migration in Drosophila wing border cells, Mad and Brk, were expressed in most tumours of our test set, as well as in most NSCLC from the validation and metastasis sets. Both were often coexpressed. Both might be important mediators of bulk migration in NSCLC. Interestingly, in NSCLC, the expression of Mad, and Brk was not under the rule of TGFβ1 [17]. Src-kinase was not phosphorylated in the majority of NSCLC cases. Nevertheless, the lack of Src activation indicates a reduction of cell cohesion and therefore an increase in bulk cell migration [17], thus fitting our observations and hypothesis.

Invadipodia formation is induced by either Rab40B or Tks5 or both [29, 30]. Coexpression of both proteins was found in half of the cases, while focal expression of Tks5 occurred in 6 additional cases. Expression was exclusively confined to carcinoma cells at the front of the tumour bulk. This is also reflected in the fact that Tks5 was expressed in two thirds of the metastasis, whereas significantly less expressed in the corresponding primary tumours. Expression of Tks5 is regulated by cortactin and neural Wiskott-Aldrich syndrome protein (N-WASP), which is frequently upregulated in NSCLC. N-WASP might also cause the upregulation of RhoA, as observed in almost all our tumour cases [29–31]. Interestingly, RhoA staining was more intense in the tumour centre compared to the invasion front, which raises some questions concerning its function: E-Cad was reported to downregulate RhoA and, in turn, reduce migration [32]. In bulk migration, E-Cad expression was retained, and RhoA expression was only reduced at the invasion site like E-Cad. RhoA in tumour centres probably have an additional function other than migration. The axis by which RhoA induce LATS, which in turn inhibit YAP/TAZ, does not function in bulk migration: Both RhoA and YAP/TAZ were expressed, the latter being present in all cases [33]. However, expression of YAP very well fit together with phosphorylation of ERK1/2, E-Cad expression, and absence of β-Catenin nuclear translocation [33].

Phosphorylated ERK1/2 was expressed in the carcinoma centre as well as at the invasion front, but less intense. As this expression was, in some cases, only present at the invasion front, ERK seems to play a role in migration, probably by interacting with other not yet identified proteins. There might exist underlying functional changes of ERK: Migrating cells usually do not proliferate, but when forming a metastatic focus, an increase in energy is required, probably reflected by ERK activation. PLCγ, another enzyme under the control of the RAS pathway, was exclusively upregulated at invasion sites and negative in tumour centres. In addition, it was highly expressed in metastasis compared with the primary tumour (invasion front), although this did not reach statistical significance. This confirms reports which already suggested that this enzyme is directly involved in migration and metastasis, probably by anaerobic decomposition of lipids [34–36]. Interestingly, PLCγ and RhoA are both induced by vascular growth factors [37]. Cytokines such as CCR7, often expressed in NSCLC, are also linked to PLCγ and the PI3K-Akt pathway [38]. PLCγ together with different integrins’ linked to the vascular endothelial growth factor family might be major drivers for angiogenesis [39].

### Factors associated with migration, homing and metastasis

Upregulation of Mad, Twist, and PLCγ in metastasis points to important functions within the metastatic site, as well. Bulk movement is most likely the preferred mode of migration at metastatic sites, too. Mad, however, might be an early mediator of migration and is thus expressed equally in primary and metastatic tumours. Tks5 was unexpectedly lost in one third of metastases. It might be that the formation of invadipodia can be regulated by other proteins. The function of PLCγ requires further investigation, as this enzyme is important for migration as well as metastasis.

Connexin43 (Con43), reported to be associated with intrapulmonary metastasis [40], was expressed in groups of carcinoma cells within the central area as well as at the invasion front. However, given the high frequency of expression in our cases, it does not seem very likely to be a marker for intrapulmonary metastasis. Con43 was equally expressed in extrathoracic metastases. It might therefore be associated with metastasis in general.

Chemokine receptors have been reported as being specifically associated with homing. CXCR4 has been reported as being responsible for metastasis to the brain [41, 42]. In our cases, the frequency of CXCR4 expression was 30%, which would fit with the reported frequency of brain metastasis, especially in AC. When comparing CXCR4 expression in the TMAmets, it was expressed in half of the primary tumours and one third of metastasis; it was expressed in low percentages of carcinoma cells, and no preferential expression in brain metastasis was seen. Therefore, CXCR4 alone most likely does not function as a mediator of brain metastasis. As it is also highly expressed in stroma cells it might point to tumour-stroma cell communication. CXCR1 has been reported as being associated with early recurrence and metastasis in low-stage adenocarcinomas [43]. As all of our cases were characterized by early recurrence and metastasis due to vascular invasion and as all carcinomas express CXCR1, our observation is consistent with this. However, CXCR1 does not seem to be directly associated with vascular invasion, as it was equally expressed in the primary tumour and at the invasion front. CXCR2 was positive in 1/3 of our cases, staining intensity was less at the invasion front; but positive reactions were higher in macrophages in all cases. Thus, CXCR2 might play a role for M2-macrophages assisting lung carcinomas to facilitate neoangiogenesis [44, 45].

Focal adhesion kinase (FAK) was expressed in only few cases and in few tumour cells. The expression of SMARCA4 (also called BRG1) was positive in all carcinomas, at equal amounts within the tumour centre and the invasion front. SMARCA4 loss has been reported in dedifferentiated carcinomas and has been associated with rapid progression. This could not be proven in our series. Despite the fact that many of the carcinomas were well to moderate differentiated, they did not lose SMARCA4 [46].

All markers identified in the study set were also found in the large cohort of carcinomas (validation set). There were some differences in the frequency of expression, especially for Mad, Tks5, PLCγ, and pERK, which might be due to the fact that, in tissue microarrays, punches are most often taken from central tumours, and therefore invasion front is not always represented and vascular invasion appears rarely, as well. The findings of frequent expression of Mad, Twist, vim, and PLCγ in metastasis confirms this interpretation. With respect to the different types, E-Cad, N-Cad, Mad, PLCγ, and Tsk5 were expressed in similar percentages in all carcinomas, while Connexin43 and Twist were more frequently expressed in SCC and LC compared to AC.

By establishing a model to study bulk migration, several aspects will be taken into account: Cell cultures cannot be used, as a low density of seeded cells will automatically induce EMT, including expression of vimentin or smooth muscle actin to reach their neighbouring cell. The cells would also lose cytokeratin and E-Cad and express N-Cad [9, 47].

## Conclusions

In conclusion, we identified bulk cancer cell migration as the predominant form in AC and SCC, whereas pure EMT is rare. In addition, we identified a mixed form composed of EMT and bulk migrations which accounts for approximately 25% of AC and SCC. Several proteins known from EMT studies have been identified, but are differently regulated in bulk migration. Twist, vimentin, fascin, Mad, Brk, Tsk5, Rab40B, ERK1/2 and PLCγ are involved in bulk cancer cell migration, but they are not under the rule of TGFβ1 (Additional file 3: Figure S1 show a schematic drawing how the different proteins might interact). The known pathways Wnt-frizzeld-βCatenin and TGFβ1, along with Snail, Slug, and ZEB1, are not involved in bulk cell migration. Bulk, complex, or hybrid migration, is placed in between EMT and epithelial complex migration representing the opposite poles [15, 48]. The homing and metastasis function of Connexin43, CXCR1, and CXCR4 require further study. Bulk migration requires an orchestrated activation of proteins to keep the cells bound to each other and to coordinate the movement. As this study was based on a retrospective analysis of lung carcinoma tissues, no functional assays were done. However, we will explore this type of migration using an in vivo experimental system.

## Additional files

Additional file 1: **Table S1.** Raw data of each case of the study set for the evaluated antibodies. Cases are numbered consecutively. (XLSX 44 kb)

Additional file 2: **Table S2.** Raw data of the evaluation of the metastasis TMA. Prim = data of the primary tumor and META = data from the corresponding metastasis; in case of several metastasis the data were pooled and a mean was calculated. In addition statistical calculations for each marker were added (student t-test for paired data). (XLSX 27 kb)

Additional file 3: **Figure S1.** Schematic cascade of migration factors. In EMT (left, orange) TGFβ activates Twist, which activates ZEB1, SNAIL and Slug; ZEB1 suppresses cytokeratin, E-Cad, and RhoA, but upregulates Vim and N-Cad. In bulk migration (green) Twist is upregulated by an unknown factor, it does neither induce ZEB1, nor SNAIL or SLUG, but likely induce YAP1; E-Cad is down- and N-Cad upregulated. YAP1 might be upregulated by N-WASP, and itself upregulates probably ERK1/2; RhoA does not block YAP1 (via LATS), and Mad/Brk probably induce Tks5 and RAB40B; vascular growth factors of the chemokine family might induce RhoA and PCLγ. (JPEG 2662 kb)

## Abbreviations

AC: Adenocarcinoma
Brk: Brinker
CdC42: Cell division cycle 42
Con43: Connexin43
CXCR1/2/4: C-X-C motif chemokine receptor 1/2/4
E-Cad: E-Cadherin
EMT: Epithelial-to-mesenchymal transition
FAK: Focal adhesion kinase
frizzled: Frizzled class receptor family
ILK: Integrin-linked kinase
LC: Large cell carcinomas
Mad: Mother against dpp
N-Cad: N-Cadherin
NSCLC: Non-small cell carcinoma
PC: Pleomorphic carcinoma
pERK1/2: Extracellular regulated MAP kinase
PLCγ: Phospholipase C gamma
pSRC: Phosphorylated SRC proto-oncogene, non-receptor tyrosine kinase
Rab40B: Member of RAS oncogene family
Rack1: Receptor of activated C kinase
RhoA: Ras homolog family member A
SARI: Basic leucine zipper ATF-like transcription factor 2
SAX: Saxophone
SCC: Squamous cell carcinoma
SCLC: Small cell carcinoma
Slug (also SNAI2): Snail family transcriptional repressor 2
SMARCA4: SWI/SNF related, matrix associated, actin dependent regulator of chromatin subfamily A member 4
TGFβ: Tumor growth factor
Tks5: Scaffold protein TKS5
TMA: Tissue microarray
TWIST: Twist family bHLH transcription factor 1
Wnt: Wnt family member
YAP1: Yes associated protein 1
ZEB1: Zinc finger E-box binding homeobox 1
ZFPL1: Zinc finger protein like 1

## References

[1] Yang J, Mani SA, Donaher JL, Ramaswamy S, Itzykson RA, Come C, Savagner P, Gitelman I, Richardson A, Weinberg RA (2004). Twist, a master regulator of morphogenesis, plays an essential role in tumor metastasis. Cell.

[2] Keshamouni VG, Michailidis G, Grasso CS, Anthwal S, Strahler JR, Walker A, Arenberg DA, Reddy RC, Akulapalli S, Thannickal VJ (2006). Differential protein expression profiling by iTRAQ-2DLC-MS/MS of lung cancer cells undergoing epithelial-mesenchymal transition reveals a migratory/invasive phenotype. J Proteome Res.

[3] Hwang MH, Cho KH, Jeong KJ, Park YY, Kim JM, Yu SL, Park CG, Mills GB, Lee HY. RCP induces Slug expression and cancer cell invasion by stabilizing beta1 integrin. Oncogene. 2017;36(8):1102-11. 10.1038/onc.2016.277 27524413

[4] Pan CM, Wang ML, Chiou SH, Chen HY, Wu CW (2016). Oncostatin M suppresses metastasis of lung adenocarcinoma by inhibiting SLUG expression through coordination of STATs and PIASs signalings. Oncotarget.

[5] Meng J, Zhang XT, Liu XL, Fan L, Li C, Sun Y, Liang XH, Wang JB, Mei QB, Zhang F (2016). WSTF promotes proliferation and invasion of lung cancer cells by inducing EMT via PI3K/Akt and IL-6/STAT3 signaling pathways. Cell Signal.

[6] Avasarala S, Van Scoyk M, Karuppusamy Rathinam MK, Zerayesus S, Zhao X, Zhang W, Pergande MR, Borgia JA, DeGregori J, Port JD (2015). PRMT1 is a novel regulator of epithelial-mesenchymal-transition in non-small cell lung cancer. J Biol Chem.

[7] Chiu LY, Hsin IL, Yang TY, Sung WW, Chi JY, Chang JT, Ko JL, Sheu GT. The ERK-ZEB1 pathway mediates epithelial-mesenchymal transition in pemetrexed resistant lung cancer cells with suppression by vinca alkaloids. Oncogene. 2016. 10.1038/onc.2016.195 PMC5241427 27270426

[8] Lin LC, Hsu SL, Wu CL, Hsueh CM (2014). TGFbeta can stimulate the p(38)/beta-catenin/PPARgamma signaling pathway to promote the EMT, invasion and migration of non-small cell lung cancer (H460 cells). Clin Exp Metastasis.

[9] Zhang HW, Wang EW, Li LX, Yi SH, Li LC, Xu FL, Wang DL, Wu YZ, Nian WQ. A regulatory loop involving miR-29c and Sp1 elevates the TGF-beta1 mediated epithelial-to-mesenchymal transition in lung cancer. Oncotarget. 2016. 10.18632/oncotarget.13137 PMC5349884 27829234

[10] Fu X, Li H, Liu C, Hu B, Li T, Wang Y (2016). Long noncoding RNA AK126698 inhibits proliferation and migration of non-small cell lung cancer cells by targeting Frizzled-8 and suppressing Wnt/beta-catenin signaling pathway. Onco Targets Ther.

[11] Friedl P, Gilmour D (2009). Collective cell migration in morphogenesis, regeneration and cancer. Nat Rev Mol Cell Biol.

[12] Lu M, Jolly MK, Onuchic J, Ben-Jacob E (2014). Toward decoding the principles of cancer metastasis circuits. Cancer Res.

[13] Jolly MK, Boareto M, Huang B, Jia D, Lu M, Ben-Jacob E, Onuchic JN, Levine H (2015). Implications of the hybrid epithelial/mesenchymal phenotype in metastasis. Front Oncol.

[14] Schliekelman MJ, Taguchi A, Zhu J, Dai X, Rodriguez J, Celiktas M, Zhang Q, Chin A, Wong CH, Wang H (2015). Molecular portraits of epithelial, mesenchymal, and hybrid states in lung adenocarcinoma and their relevance to survival. Cancer Res.

[15] Boareto M, Jolly MK, Goldman A, Pietila M, Mani SA, Sengupta S, Ben-Jacob E, Levine H, Onuchic JN. Notch-Jagged signalling can give rise to clusters of cells exhibiting a hybrid epithelial/mesenchymal phenotype. J R Soc Interface. 2016;13(118). 10.1098/rsif.2015.1106. 10.1098/rsif.2015.1106 PMC4892257 27170649

[16] Gabor S, Renner H, Popper H, Anegg U, Sankin O, Matzi V, Lindenmann J, Smolle Juttner FM (2004). Invasion of blood vessels as significant prognostic factor in radically resected T1-3N0M0 non-small-cell lung cancer. Eur J Cardiothorac Surg.

[17] Luo J, Zuo J, Wu J, Wan P, Kang D, Xiang C, Zhu H, Chen J (2015). In vivo RNAi screen identifies candidate signaling genes required for collective cell migration in Drosophila ovary. Sci China Life Sci.

[18] Huang B, Jolly MK, Lu M, Tsarfaty I, Ben-Jacob E, Onuchic JN (2015). Modeling the transitions between collective and solitary migration phenotypes in cancer metastasis. Sci Rep.

[19] Pirozzi G, Tirino V, Camerlingo R, Franco R, La Rocca A, Liguori E, Martucci N, Paino F, Normanno N, Rocco G (2011). Epithelial to mesenchymal transition by TGFbeta-1 induction increases stemness characteristics in primary non small cell lung cancer cell line. PLoS One.

[20] Gemmill RM, Roche J, Potiron VA, Nasarre P, Mitas M, Coldren CD, Helfrich BA, Garrett-Mayer E, Bunn PA, Drabkin HA (2011). ZEB1-responsive genes in non-small cell lung cancer. Cancer Lett.

[21] Smit MA, Geiger TR, Song JY, Gitelman I, Peeper DS (2009). A twist-snail axis critical for TrkB-induced epithelial-mesenchymal transition-like transformation, anoikis resistance, and metastasis. Mol Cell Biol.

[22] Wang Y, Liu J, Ying X, Lin PC, Zhou BP (2016). Twist-mediated epithelial-mesenchymal transition promotes breast tumor cell invasion via inhibition of hippo pathway. Sci Rep.

[23] Wang C, Su Y, Zhang L, Wang M, You J, Zhao X, Zhang Z, Liu J, Hao X (2012). The function of SARI in modulating epithelial-mesenchymal transition and lung adenocarcinoma metastasis. PLoS One.

[24] Olins AL, Herrmann H, Lichter P, Olins DE (2000). Retinoic acid differentiation of HL-60 cells promotes cytoskeletal polarization. Exp Cell Res.

[25] Messica Y, Laser-Azogui A, Volberg T, Elisha Y, Lysakovskaia K, Eils R, Gladilin E, Geiger B, Beck R (2017). The role of vimentin in regulating cell invasive migration in dense cultures of breast carcinoma cells. Nano Lett.

[26] Van Audenhove I, Denert M, Boucherie C, Pieters L, Cornelissen M, Gettemans J (2016). Fascin rigidity and L-plastin flexibility cooperate in cancer cell Invadopodia and Filopodia. J Biol Chem.

[27] Lin S, Lu S, Mulaj M, Fang B, Keeley T, Wan L, Hao J, Muschol M, Sun J, Yang S (2016). Monoubiquitination inhibits the actin bundling activity of fascin. J Biol Chem.

[28] Liang Z, Wang Y, Shen Z, Teng X, Li X, Li C, Wu W, Zhou Z, Wang Z (2016). Fascin 1 promoted the growth and migration of non-small cell lung cancer cells by activating YAP/TEAD signaling. Tumour Biol.

[29] Seals DF, Azucena EF, Pass I, Tesfay L, Gordon R, Woodrow M, Resau JH, Courtneidge SA (2005). The adaptor protein Tks5/Fish is required for podosome formation and function, and for the protease-driven invasion of cancer cells. Cancer Cell.

[30] Jacob A, Linklater E, Bayless BA, Lyons T, Prekeris R (2016). The role and regulation of Rab40b-Tks5 complex during invadopodia formation and cancer cell invasion. J Cell Sci.

[31] Crimaldi L, Courtneidge SA, Gimona M (2009). Tks5 recruits AFAP-110, p190RhoGAP, and cortactin for podosome formation. Exp Cell Res.

[32] Asnaghi L, Vass WC, Quadri R, Day PM, Qian X, Braverman R, Papageorge AG, Lowy DR (2010). E-cadherin negatively regulates neoplastic growth in non-small cell lung cancer: role of Rho GTPases. Oncogene.

[33] Sun D, Li X, He Y, Li W, Wang Y, Wang H, Jiang S, Xin Y. YAP1 enhances cell proliferation, migration, and invasion of gastric cancer in vitro and in vivo. Oncotarget. 2016. 10.18632/oncotarget.13188 PMC5348376 27835600

[34] Jones NP, Peak J, Brader S, Eccles SA, Katan M (2005). PLCgamma1 is essential for early events in integrin signalling required for cell motility. J Cell Sci.

[35] Di Blasio L, Gagliardi PA, Puliafito A, Primo L. Serine/Threonine kinase 3-phosphoinositide-dependent protein Kinase-1 (PDK1) as a key regulator of cell migration and cancer dissemination. Cancers (Basel). 2017;9(3). 10.3390/cancers9030025 PMC5366820 28287465

[36] Phillips-Mason PJ, Kaur H, Burden-Gulley SM, Craig SE, Brady-Kalnay SM (2011). Identification of phospholipase C gamma1 as a protein tyrosine phosphatase mu substrate that regulates cell migration. J Cell Biochem.

[37] Wang J, Taba Y, Pang J, Yin G, Yan C, Berk BC (2009). GIT1 mediates VEGF-induced podosome formation in endothelial cells: critical role for PLCgamma. Arterioscler Thromb Vasc Biol.

[38] Li Y, Qiu X, Zhang S, Zhang Q, Wang E (2009). Hypoxia induced CCR7 expression via HIF-1alpha and HIF-2alpha correlates with migration and invasion in lung cancer cells. Cancer Biol Ther.

[39] Wong C, Jin ZG (2005). Protein kinase C-dependent protein kinase D activation modulates ERK signal pathway and endothelial cell proliferation by vascular endothelial growth factor. J Biol Chem.

[40] Elzarrad MK, Haroon A, Willecke K, Dobrowolski R, Gillespie MN, Al-Mehdi AB (2008). Connexin-43 upregulation in micrometastases and tumor vasculature and its role in tumor cell attachment to pulmonary endothelium. BMC Med.

[41] Wang L, Wang Z, Liu X, Liu F (2014). High-level C-X-C chemokine receptor type 4 expression correlates with brain-specific metastasis following complete resection of non-small cell lung cancer. Oncol Lett.

[42] Salmaggi A, Maderna E, Calatozzolo C, Gaviani P, Canazza A, Milanesi I, Silvani A, DiMeco F, Carbone A, Pollo B (2009). CXCL12, CXCR4 and CXCR7 expression in brain metastases. Cancer Biol Ther.

[43] Iwakiri S, Mino N, Takahashi T, Sonobe M, Nagai S, Okubo K, Wada H, Date H, Miyahara R (2009). Higher expression of chemokine receptor CXCR7 is linked to early and metastatic recurrence in pathological stage I nonsmall cell lung cancer. Cancer.

[44] Liu Q, Li A, Tian Y, Wu JD, Liu Y, Li T, Chen Y, Han X, Wu K (2016). The CXCL8-CXCR1/2 pathways in cancer. Cytokine Growth Factor Rev.

[45] Saintigny P, Massarelli E, Lin S, Ahn YH, Chen Y, Goswami S, Erez B, O'Reilly MS, Liu D, Lee JJ (2013). CXCR2 expression in tumor cells is a poor prognostic factor and promotes invasion and metastasis in lung adenocarcinoma. Cancer Res.

[46] Le Loarer F, Watson S, Pierron G, de Montpreville VT, Ballet S, Firmin N, Auguste A, Pissaloux D, Boyault S, Paindavoine S (2015). SMARCA4 inactivation defines a group of undifferentiated thoracic malignancies transcriptionally related to BAF-deficient sarcomas. Nat Genet.

[47] Kuner R, Muley T, Meister M, Ruschhaupt M, Buness A, Xu EC, Schnabel P, Warth A, Poustka A, Sultmann H (2009). Global gene expression analysis reveals specific patterns of cell junctions in non-small cell lung cancer subtypes. Lung Cancer.

[48] Shamir ER, Coutinho K, Georgess D, Auer M, Ewald AJ (2016). Twist1-positive epithelial cells retain adhesive and proliferative capacity throughout dissemination. Biology Open.

