# Supplementary figures and images for: Bulk tumour cell migration in lung carcinomas might be more common than epithelial-mesenchymal transition and be differently regulated
Source: BMC Cancer. 2018 Jul 6;18:717. doi: 10.1186/s12885-018-4640-y (PMC6034257; doi:10.1186/s12885-018-4640-y)

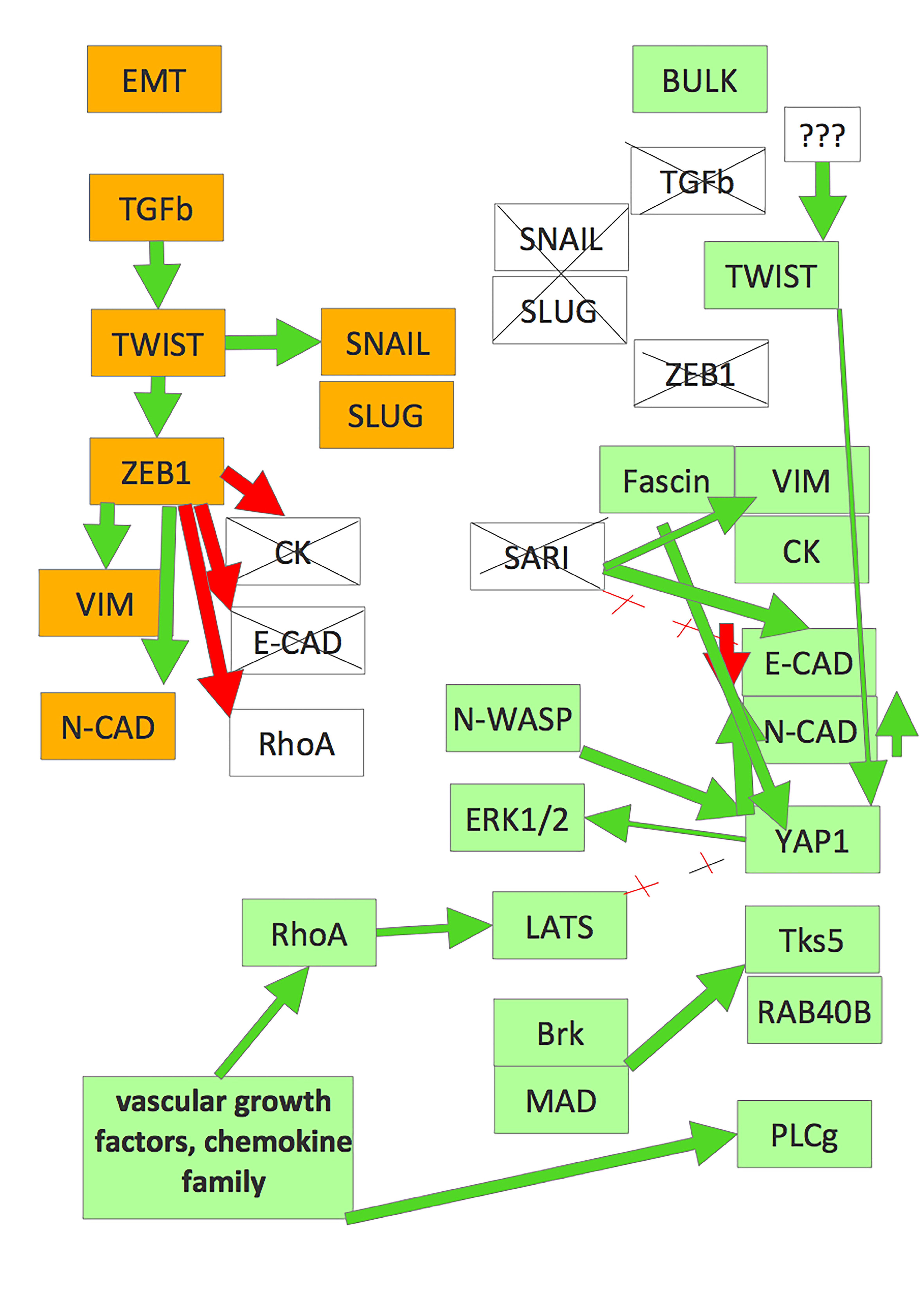

Supplement: Supplementary file 3 — Figure S1. Schematic cascade of migration factors. In EMT (left, orange) TGFβ activates Twist, which activates ZEB1, SNAIL and Slug; ZEB1 suppresses cytokeratin, E-Cad, and RhoA, but upregulates Vim and N-Cad. In bulk migration (green) Twist is upregulated by an unknown factor, it does neither induce ZEB1, nor SNAIL or SLUG, but likely induce YAP1; E-Cad is down- and N-Cad upregulated. YAP1 might be upregulated by N-WASP, and itself upregulates probably ERK1/2; RhoA does not block YAP1 (via LATS), and Mad/Brk probably induce Tks5 and RAB40B; vascular growth factors of the chemokine family might induce RhoA and PCLγ. (JPEG 2662 kb) [file 12885_2018_4640_MOESM3_ESM.jpeg]
